# Analysis of Vitamin D and VDR Expression in Women with Advanced Endometriosis: A Case–Control Study in Thailand

**DOI:** 10.3390/biomedicines13071605

**Published:** 2025-06-30

**Authors:** Vitet Layanun, Woraluk Somboonporn, Pinya Aupongkaroon, Pilaiwan Kleebkaow, Nipon Chaisuriya, Naree Pluthikarmpae

**Affiliations:** 1 Department of Obstetrics and Gynecology, Faculty of Medicine, Khon Kaen University, Khon Kaen 40002, Thailand; vitela@kku.ac.th (V.L.); pinya1990@hotmail.com (P.A.); kpilai@kku.ac.th (P.K.); 2 Department of Pathology, Faculty of Medicine, Khon Kaen University, Khon Kaen 40002, Thailand; 3 Department of Pathology, Suranaree University of Technology Hospital, Nakhon Ratchasima 30000, Thailand; naree.pl@g.sut.ac.th

**Keywords:** vitamin D, endometriosis, vitamin D receptor, immunohistochemistry, H-score

## Abstract

**Background:** Vitamin D has anti-inflammatory and immunomodulatory properties that may influence the pathophysiology of endometriosis. This study investigated the association between vitamin D levels and endometriosis, and vitamin D receptor (VDR) expression in endometriotic tissue. **Methods:** A cross-sectional study was conducted involving 36 patients with endometriosis and 72 healthy control women, matched for age and BMI. Serum 25-hydroxyvitamin D levels were measured and categorized into four statuses (normal, insufficiency, deficiency, and severe deficiency). Endometriotic tissue samples were examined for VDR expression using immunohistochemistry and qualitatively quantified using histo-scores (H-scores). Endometriosis severity was assessed using the revised criteria of the American Society for Reproductive Medicine (rASRM). **Results:** No statistically significant difference in vitamin D levels between the groups (20.45 vs. 21.10 ng/dL, *p* = 0.190) was observed, even after adjusting for residence, body sunscreen use, pregnancy, and contraceptive use. VDR expression exhibited significantly higher H-scores in endometriotic epithelial cells than in stromal cells (209.51 vs. 73.32; *p* < 0.001). Additionally, the VDR H-score in both cell compartments showed no significant difference according to vitamin D status. No statistically significant association was found between vitamin D levels, VDR expression, or disease severity. The odds of severe endometriosis were 2.17 (95% CI: 0.14–33.80) for vitamin D insufficiency and 4.33 (95% CI: 0.24–115.67) for deficiency compared with normal vitamin D. **Conclusions:** There was no statistically significant association between vitamin D levels and endometriosis and VDR.

## 1. Introduction

Endometriosis is a chronic inflammatory condition characterized by the growth of functional endometrial tissue outside the uterine cavity [1]. Ectopic endometrial implants can invade pelvic organs, leading to chronic pelvic pain, dysmenorrhea, and infertility [2,3]. Endometriosis significantly impacts global health, affecting approximately 5–10% of reproductive-age women (176 million women worldwide). The peak incidence occurs in women aged 25–35 [2,4,5]. Endometriosis imposes significant quality-of-life and economic costs; healthcare expenditures for affected women are approximately double those of unaffected women, establishing this disease as a public health concern requiring improved management [6,7]. The pathogenesis of endometriosis is complex and multifactorial. Retrograde menstruation is a generally recognized origin theory; however, disease development involves a complex interplay of genetic, immunological, and environmental factors [2,8]. Endometriosis creates a chronic pro-inflammatory peritoneal environment, with oxidative stress and immune dysregulation contributing to lesion adhesion, invasion, proliferation, and progression [4,9].

Vitamin D has become a significant focus of research in endometriosis due to its immunomodulatory and anti-inflammatory properties [10,11,12]. Vitamin D is a fat-soluble steroid hormone that regulates gene expression and immune function [4]. Vitamin D deficiency has been linked to various diseases, including autoimmune and reproductive disorders [13,14]. Vitamin D may influence the development and progression of endometriosis through its immunomodulatory, anti-inflammatory, and anti-proliferative properties. It regulates the growth and inflammatory response of endometriotic cells, potentially by modulating key mediators such as interleukin-6 (IL-6), interleukin-8 (IL-8), prostaglandins, and matrix metalloproteinases [15]. Observational studies reporting lower serum vitamin D levels in women with endometriosis suggest that vitamin D deficiency may contribute to the excessive cellular proliferation and persistent inflammation characteristic of this disease [16]. Several studies have investigated the relationship between vitamin D status and endometriosis; however, their findings have been inconsistent. A recent cross-sectional analysis of the U.S. National Health and Nutrition Examination Survey data reported that women with sufficient 25(OH)D levels (≥20 ng/mL) had a lower prevalence of endometriosis, suggesting a potential protective effect of vitamin D [6]. In contrast, other studies have found no significant difference in serum vitamin D levels between women with endometriosis and controls [17,18]. The local vitamin D system in endometriotic tissue may be altered; endometriotic tissue exhibits higher expression of the vitamin D receptor (VDR) than the eutopic endometrium, and increased expression of the activating enzyme 1α-hydroxylase has been observed [11]. A recent study used the immunoreactivity score (IRS) to evaluate immunohistochemical (IHC) staining for VDR, comparing three phases of normal endometrium in premenopausal women with endometriosis. The results indicated an upregulation of the nucleus in epithelial cells from endometriosis, with no significant differences observed across the three phases in the cytoplasm. However, that study did not assess the vitamin D status of the patients [19]. Genetic studies on vitamin D pathways have yielded mixed results; polymorphisms in VDR and vitamin D-binding protein (VDBP) genes were not significantly associated with endometriosis susceptibility in some populations [20,21,22]. A recent systematic review highlighted a possible link between low vitamin D levels and endometriosis [23]; however, the overall evidence remains inconclusive.

In Thailand, vitamin D deficiency is prevalent (approximately 60% of working-age adults) [24,25]. Given the high local burden of endometriosis at our tertiary care center, it is important to clarify the relationship between vitamin D levels and endometriosis in our population. Moreover, given the limited evidence regarding the relationship between vitamin D levels and VDR expression in endometriosis, investigating VDR expression in conjunction with circulating vitamin D levels is essential to advance our understanding of this area and elucidate the potential underlying mechanisms. We aimed to examine the association between vitamin D levels and endometriosis and evaluated VDR expression in endometriotic lesions to explore whether circulating vitamin D levels or tissue VDR expression correlated with the severity of endometriosis. We hypothesized that women with endometriosis would have different vitamin D levels compared to controls and that vitamin D levels would correlate with VDR expression in endometriotic lesions. Additionally, vitamin D levels and VDR expression may correlate with endometriosis severity. Therefore, this study aimed to enhance our understanding of the role of vitamin D and the vitamin D receptor (VDR) in the pathogenesis of endometriosis, which may inform future preventive and therapeutic strategies.

## 2. Materials and Methods

### 2.1. Study Design and Participants

This cross-sectional prospective matching study was conducted at Srinagarind Hospital, Faculty of Medicine, Khon Kaen University (Thailand), between May 2024 and December 2024. Women in both groups were aged 18 years or older. The endometriosis group comprised 36 women scheduled for surgical treatment, either laparoscopy or laparotomy. The healthy control group comprised 72 women who visited the outpatient department of the hospital for routine health check-ups. We implemented an exact matching procedure to select controls for each endometriosis case at a 1:2 ratio of cases to controls. Controls were matched for age and body mass index (BMI). The sample size calculation was performed based on the results from Cho et al. 2019 [21] (α = 0.05, β = 0.20, r = 2, T = 2, v = 6.95, *ρ* = 0.01, and σ = 16) using the statistical formula: n/group = (Z_1−α/2_ + Z_1−β_)^2^ σ^2^(r + 1) [1 + (T − 1)*ρ*]/v^2^rT, with an additional 10% dropout included. All participants provided written informed consent prior to their enrollment. The study protocol was approved by the Khon Kaen University Center for Ethics in Human Research (reference no. HE661574, approved 23 January 2024). This study was registered at clinicaltrials.gov as NCT06289257 on 20 February 2024.

Inclusion Criteria for Endometriosis: Women diagnosed with endometriosis scheduled for surgery at Srinagarind Hospital without any underlying disease. Inclusion criteria for Controls: Outpatient healthy women with no underlying disease, no history of gynecological diseases, and no treatment for gynecological conditions associated with pelvic pain, infection, or mass in the pelvis. Additionally, the most recent pelvic and ultrasonographic examinations in the previous year should be normal. Overall Exclusion Criteria: No history of vitamin D intake within 3 months before enrollment, chronic infection, autoimmune disease treatment, polycystic ovary syndrome (PCOS), bone disease, medications that affect bones, irregular menstruation or amenorrhea for more than 3 months to 1 year, unless due to a contraceptive method.

### 2.2. Data Collection and Measurements

Baseline demographic and lifestyle data were collected using a structured interview questionnaire. The information obtained included age, BMI, partner status, pregnancy history, contraception, surgical history, and alcohol consumption. Sun exposure was assessed in terms of occupational environment (<50% vs. ≥50% of working time outdoors), average weekly sun exposure during working days, sunscreen use, outdoor exercise, and place of residence (rural vs. urban). The clinical data for the endometriosis group included pre-operative medical treatment for endometriosis, recorded pain scores, and surgeon-assessed disease stage. 

Serum vitamin D levels were measured in all participants. Blood was collected from the endometriosis group within 31 days before surgery. The control group was obtained during their check-up visits within the same period as the matched participants. Serum 25(OH)D was measured using an Elecsys vitamin D total kit with a Cobas e801 module (Roche Diagnostics, Mannheim, Germany). Vitamin D status was categorized as normal (≥30 ng/mL), insufficient (20–29.99 ng/mL), deficient (12–19.99 ng/mL), or severely deficient (<12 ng/mL) [13,26,27,28]. Further laboratory tests included serum calcium, magnesium, phosphorus, albumin, creatinine, and hepatic transaminase levels.

Endometriosis severity was graded according to the Revised American Society for Reproductive Medicine (rASRM) classification based on operative findings, including lesion size, extent, adhesions, disease lesions, and cystic fluid characteristics, by a gynecologist who was not involved in the study. Stages I–IV were determined by scores as follows: 1–5 as stage I (minimal), 6–15 as stage II (mild), 16–40 as stage III, and >40 as stage IV (severe) [29].

Tissue samples were collected during surgery from the endometriosis group. Two independent certified pathologists evaluated hematoxylin and eosin (H&E)-stained slides to assess the adequacy of endometriotic foci. Formalin-fixed, paraffin-embedded tissues were selected, and sections were prepared at a thickness of 4–5 µm. Appendix or small bowel tissue was used as an external positive control. A mouse monoclonal antibody clone D-6 (Santa Cruz Biotechnology, Dallas, TX, USA) directed against VDR was used at a dilution of 1:200 in a Ventana Ultra autostainer. H&E and immunohistochemistry slides were digitized using a NanoZoomer S360 digital slide scanner (Hamamatsu Photonics, Hamamatsu, Japan). The digital slide files were subsequently viewed using NDV View version 2.8.24 (Hamamatsu Photonics, Hamamatsu, Japan).

**Figure 1:**
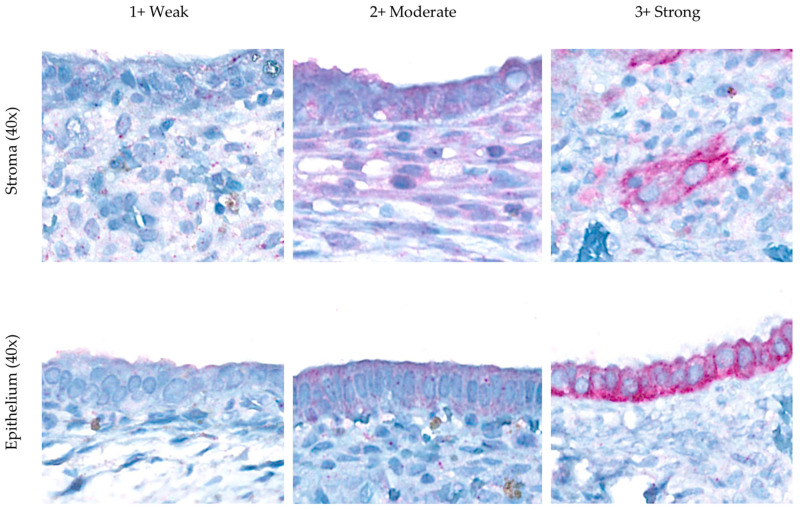
Immunohistochemical expression of VDR in the cytoplasm of the endometriosis group.

VDR expression, scored using the histo-score (H-score) from IHC-stained slides, was interpreted by two independent pathologists. The pathologists graded the staining intensity into four categories: negative (0), weak (1+), moderate (2+), and strong (3+), and described the percentage of the area stained (Figure 1). The H-score had an analytical range of 0–300 and was calculated using the following formula: H-score = [(0 × P_0_) + (1 × P_1_) + (2 × P_2_) + (3 × P_3_)]. This formula uses the percentage (P) of cells (ranging from 0% to 100%), where P0 represents the proportion of negative cells, P_1_ represents the proportion of weakly stained cells, P_2_ represents the proportion of moderately stained cells, and P_3_ represents the proportion of strongly stained cells [30]. The intraclass correlation coefficient (ICC) score between the two pathologists for both stroma and epithelium was 0.56, indicating poor reliability in stroma but excellent reliability in epithelial component expression. The average H-score of the stromal and epithelial cells was used for the analysis.

### 2.3. Statistical Analysis

All statistical analyses were performed using STATA version 18.5 (StataCorp, College Station, TX, USA). Categorical variables are expressed as frequencies (%). Continuous variables were assessed for normality using the Shapiro–Wilk test and are shown as mean ± SD if normally distributed, or median [IQR] if not. Matched comparisons between the endometriosis and control groups were performed using conditional logistic regression analyses. Unmatched categorical comparisons were performed using Fisher’s exact test, and odds ratios (ORs) and 95% confidence intervals (CIs) were estimated using conditional logistic regression. As appropriate, continuous variables were compared using paired or independent *t*-tests, Kruskal–Wallis tests, and one-way ANOVA. Unmatched continuous differences were assessed using linear regression analysis. Correlations were evaluated using Spearman’s rank correlation coefficients (95% CI). Multivariable analyses were performed for adjusted matched data using generalized estimating equations (GEE) with an exchangeable correlation and conditional logistic regression. Furthermore, controls were matched only by age and BMI, but other confounders (e.g., sun exposure and diet) were adjusted post hoc. A two-sided *p*-value < 0.05 was considered statistically significant.

## 3. Results

Thirty-six women with endometriosis and seventy-two matched healthy controls were included in the analysis. 

Participant Characteristics: The mean age was comparable between the endometriosis and control groups (33.36 ± 5.41 vs. 33.35 ± 5.63 years, *p* = 0.938). The median body mass index (BMI) was also comparable (21.76 [19.93–23.19] vs. 21.50 [19.24–23.87] kg/m^2^, *p* = 0.927), with both groups having a majority of participants in the normal BMI range (63.89%) and equal proportions in the underweight, overweight, and obesity type I categories. A similar percentage of participants in each group lived with their partner(s). 

Prior pregnancy history was less common in the endometriosis group (25.00% vs. 47.22%, *p* = 0.028), whereas the history of abortion showed no significant difference (*p* = 0.344; Table 1). Contraceptive use patterns differed significantly between groups (*p* = 0.041); however, medical treatments with contraceptive effects in the endometriosis group were not included in this study. 

There was a trend toward a higher proportion of prior lower abdominal surgery in the endometriosis group (30.6% vs. 15.3% had at least one lower abdominal or pelvic surgery) (Table S1). 

Most of the participants had college degrees. All participants reported indoor occupations (100% in both groups worked outdoors for <50% of the time). 

Total sun exposure did not differ significantly, with a median of 115 min weekly for patients with endometriosis compared to 75 min for controls (*p* = 0.304). Direct sunlight exposure was also similar (74.25 vs. 45.00 min, *p* = 0.133). The proportion of each group working night shifts did not differ significantly. Sunscreen use was similar across all groups. The most commonly used high-SPF facial and body sunscreen products were comparable (Table 1). Outdoor exercise was similar in both groups; most did not engage in regular outdoor exercise, but the evening was the most common time for engaging in exercise (Table S1).

Both groups had similar dietary habits. All participants consumed meat, and most consumed dairy products (58.33% vs. 66.67%, *p* = 0.385). The use of vitamin supplements was low and similar across both groups, with approximately one-quarter of each group reporting vitamin supplementation (Table 1). 

Rural vs. urban residency showed a significant baseline difference (*p* = 0.003). More patients with endometriosis lived in rural areas (52.78% vs. 20.83%), whereas an urban predominance was observed in the control group (79.17% vs. 47.22%) (Table 1). No statistically significant differences were observed in any of the measured biochemical parameters. 

Treatment prior to surgery in the endometriosis group was heterogeneous, including NSAIDs (36.11%), GnRH agonists (19.44%), dienogest (19.44%), DMPA (16.67%), and combined oral contraceptives (2.78%), while 5.56% of patients received no medication. The median interval from treatment initiation to surgery was 9.00 [5.00–12.00] months. Patient-reported outcomes demonstrated a median pain VAS of 5.40 [2.60–8.60] cm and a mean treatment satisfaction VAS of 6.92 ± 2.54 cm (Table S6).

Significant differences were observed according to deficiency in the hypovitaminosis D subgroup (≥20 vs. <20 ng/mL) in the history of lower abdominal or pelvic surgery (*p* = 0.016) and sunscreen use (*p* < 0.001) (Table S1). Matched univariable analysis showed that contraceptive users had 85% lower odds of endometriosis than non-users (OR 0.15, 95% CI 0.05–0.49; *p* = 0.002). Pregnancy and urban residency were protective factors, with an OR of 0.41 and 0.20, respectively. Higher education and body sunscreen use each showed a non-significant trend toward an increased risk (OR 3.04, 95% CI 0.72–12.82; *p* = 0.130, and OR 2.32, 95% CI 0.91–5.94; *p* = 0.079, respectively) (Table S2).

The median serum 25(OH)D levels were not significantly lower in the endometriosis group than in the control group (20.45 [16.10–25.10] vs. 21.10 [18.20–26.70] ng/mL) (Table 2). The unadjusted difference between the groups was −1.67 ng/mL (95% CI, −4.16–0.83), which was not statistically significant (*p* = 0.190). After adjusting for residence and body sunscreen use (Model 1), the endometriosis group exhibited significantly lower vitamin D levels (−2.90 ng/mL; 95% CI, −5.26 to −0.54; *p* = 0.016). Further adjustments for pregnancy and contraception (Model 2) and residence, body sunscreen use, pregnancy, and contraception (Model 3) showed non-significant differences. The overall distribution of participants did not differ between the vitamin D status subgroups (*p* = 0.842). Using normal vitamin D levels (≥30.00 ng/mL) as the reference, hypovitaminosis D (<30.00 ng/mL) was not significantly associated with the risk of endometriosis (OR 1.84; 95% CI, 0.54–6.36). When vitamin D status was reclassified as deficient (<20.00 ng/mL) vs. non-deficient (≥20.00 ng/mL, reference), the crude OR was 1.12 (95% CI, 0.45–2.77), and the adjusted ORs were 1.99 (95% CI, 0.63–6.35) in Model 1, 0.72 (95% CI, 0.24–2.13) in Model 2, and 1.28 (95% CI, 0.39–4.16) in Model 3; neither models reached statistical significance (Table 3). However, women with endometriosis were less likely to have normal vitamin D levels, and severe vitamin D deficiency was observed solely in patients with endometriosis (Table S4).

In the endometriosis group, VDR immunohistochemical expression was observed solely in the cytoplasm of stromal and epithelial cells, with no nuclear staining detected. Expression was consistently higher in epithelial cells than in stromal cells in each subgroup of vitamin D status, with a mean H-score of 209.51 ± 65.26 vs. 73.32 ± 36.00 (*p* < 0.001). The mean stromal and epithelial H-scores were not significantly different across the vitamin D subgroups (*p* = 0.876 and *p* = 0.908, respectively) (Table 4).

Among women with endometriosis stages III and IV, there was no statistically significant association between vitamin D status and disease severity (*p* = 0.390). The mean vitamin D levels showed no significant difference between stages III and IV (22.55 ± 5.83 vs. 20.73 ± 6.24, *p* = 0.584). The distribution of the rASRM stage did not differ significantly according to vitamin D status (*p* = 0.390), and no statistically significant correlation was observed between the vitamin D subgroup and disease severity stage (Table 5).

**Figure 2:**
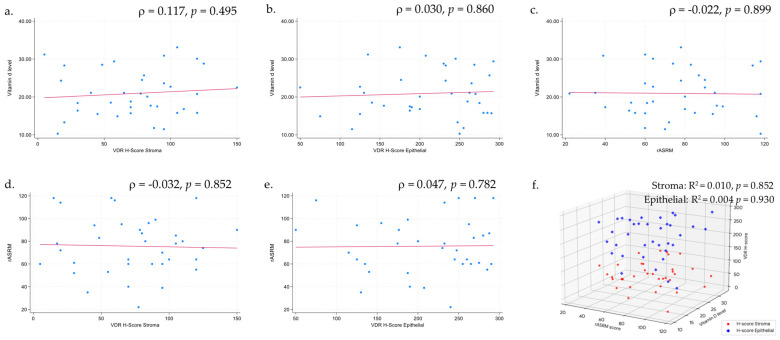
Correlations between serum vitamin D level, VDR expression, and rASRM score. Spearman’s rho analysis reveal no significant linear relationships among these variables, and scatterplots (**a**–**e**) also show no noticeable patterns consistent with a lack of association. Multiple linear regression (**f**) show no significant associations between serum 25(OH)D levels and rASRM scores or VDR H-scores (stromal or epithelial).

Spearman’s rho correlation analyses did not demonstrate any significant linear relationships between vitamin D level, VDR expression (stromal or epithelial H-scores), and rASRM score (all *p* > 0.05). Similarly, the corresponding scatter plots (Figure 2a–e) show no discernible patterns in the data. Consistent with these results, multivariable linear regression confirmed the absence of significant associations among these continuous variables (all *p* > 0.05) (Figure 2f).

## 4. Discussion

### 4.1. Serum Vitamin D Levels in Women with Endometriosis Compared to Controls

Contrary to our hypothesis, we observed no statistically significant difference in serum total 25(OH)D levels between women with endometriosis and those in the control group. After adjusting for factors associated with endometriosis (pregnancy and contraception; Table 2), the vitamin D levels did not differ between the groups. Only after adjusting for residence and body sunscreen use, factors related to vitamin D levels, were there any significant differences. This finding is consistent with previous studies that reported no significant difference in vitamin D levels between the two groups. For instance, Buggio et al. (2019) found comparable serum vitamin D levels among Italian women diagnosed with ovarian or deep infiltrating endometriosis and healthy controls (17.90 ± 7.00 vs. 18.40 ± 7.60 ng/mL; *p* = 0.460) [17]. Similarly, Somigliana et al. reported no significant differences in vitamin D levels in an Italian cohort [18]. However, our findings contrast with those of several studies conducted in other populations, which reported significantly lower vitamin D levels in women with endometriosis. Studies from Korea, Iran, Russia, and Italy have found that patients with endometriosis have significantly lower serum 25(OH)D levels than controls (*p* < 0.05) [21,31,32,33]. The inconsistent results across studies may be attributed to variations in study design, population characteristics, and environmental factors [4,23,34,35,36]. The relatively low vitamin D levels observed in the control group may be explained by the high proportion of urban residents (79.17%); however, in the endometriosis group, only 47.22% of the patients resided in urban areas. This pattern aligns with the findings of Siwamogsatham et al. (2015), who reported that 45.20% of the Thai population exhibits vitamin D insufficiency (defined as a level of <30 ng/mL), with a higher prevalence among women, younger individuals, and those living in urban areas [37]. Consequently, the lower vitamin D levels among urban residents may partly explain the non-significant differences in vitamin D levels observed between the endometriosis and control groups. 

The variability in the results across the adjusted models may be attributed to differences in the direction and strength of the associations between the covariates and vitamin D levels. In our study, lower vitamin D levels were more prevalent among participants living in urban areas and those using sunscreen, who predominantly comprised the control group. Consequently, urban residence and sunscreen use were significant confounders. When we adjusted for the two confounders in Model 1, the positive association between vitamin D levels and study outcomes became more apparent. Models 2 and 3 included additional adjustments for factors known to be associated with endometriosis. These adjustments may have attenuated the observed differences in vitamin D levels between the endometriosis and control groups. These findings underscore the need for further research to investigate the relationship between vitamin D status and endometriosis, with careful consideration of potential confounders such as urban residence and sunscreen use.

In either the crude or adjusted models, no statistically significant association was observed when vitamin D levels were classified into four categories: normal, insufficient, deficient, and severely deficient. Similarly, our analyses did not show statistically significant odds ratios (ORs). Specifically, hypovitaminosis D (<30 ng/mL) was not associated with an increased risk of endometriosis (OR = 1.84, 95% CI: 0.54–6.36). Furthermore, when we re-analyzed the data using the adjusted odds ratio (AOR) for vitamin D deficiency defined as <20 ng/mL, consistent with the criteria used in previous studies, the direction of the association remained unchanged (Table 3). Notably, 8.33% of women with endometriosis were classified as severely deficient, whereas none were classified as severely deficient in the control group. Contrary to a study of 110 Iranian women by Delbandi et al. (2021), a higher risk of endometriosis was reported in women with serum 25(OH)D < 20 ng/mL (OR = 2.70, 95% CI 1.24–5.80; *p* = 0.01) [31]. In the most extensive and recent NHANES analysis of 3232 U.S. women, those with vitamin D levels ≥20 ng/mL had 27% lower odds of endometriosis than those with deficient levels (adjusted OR = 0.73, 95% CI 0.54–0.97; *p* < 0.001) [6].

### 4.2. Vitamin D Receptor (VDR) Expression

IHC analysis of VDR expression in rASRM stage III–IV endometriotic lesions demonstrated that VDR immunoreactivity was confined to the cytoplasm of both stromal and epithelial cells (Figure 1). This strictly cytoplasmic pattern diverges from the predominantly nuclear or mixed nuclear–cytoplasmic staining reported in earlier studies [11,16,19]. Although only cytoplasmic VDR staining was observed, this does not preclude vitamin D/VDR biological activity. As cytoplasmic VDR serves as a reservoir for ligand-dependent genomic activation and a platform for non-genomic signaling [38], it translocates to the nucleus after ligand binding. This predominance of cytoplasmic staining may be explained by our use of a C-terminal antibody clone (against amino acids 344–424 of the human VDR), low-pH citrate retrieval that may insufficiently unmask nuclear epitopes for this clone, restriction of the sample set to rASRM stage III–IV lesions, and predominance of hypovitaminosis D cases, all of which favor cytoplasmic retention of unliganded or phosphorylated VDR capable of both genomic shuttling and rapid non-genomic signaling [39,40]. The discrepancy between the differences in VDR expression across compartments in previous studies may be attributable to the use of different antibody clones in each study. Therefore, qualitative and quantitative H-scoring of cytoplasmic expression remains biologically informative, even in the absence of a nuclear signal. 

VDR H-scores differed significantly between stroma and epithelium, with median stromal H-scores being significantly lower than the epithelium. In a previous study in which VDR staining was limited to the nuclear compartment (a positive/negative qualified assessment), the endometriotic epithelium showed significantly stronger VDR expression than the stroma and normal ovarian cells beneath it [11,41]. 

### 4.3. Serum Vitamin D Levels and VDR Expression

According to vitamin D status, the difference in both stromal and epithelial H-scores was not significant, and the association between vitamin D level and H-score was not significant (Table 4, Figure 2a,b). To the best of our knowledge, no prior study has simultaneously investigated vitamin D status, vitamin D receptor (VDR) expression, and their relationship with endometriosis. Nevertheless, emerging evidence suggests a broadly protective role for vitamin D/VDR signaling in female reproductive tissues. Previous research examining vitamin D status and VDR expression in relation to other gynecologic diseases has found that high systemic 25(OH)D levels and strong tissue VDR expression are associated with a reduced risk of gynecologic malignancies, including ovarian and endometrial cancer [42,43,44].

### 4.4. Serum Vitamin D Level and Endometriosis Severity 

All 36 participants had a moderate-to-severe disease: 4 (11.1%) in stage III and 32 (88.9%) in stage IV. Vitamin D levels did not differ significantly between stages III and IV (*p* = 0.584). The limited range of endometriosis severity within the study population likely hindered the ability to produce reliable estimates, resulting in an insignificant association between vitamin D levels and rASRM scores (*ρ* = −0.022, *p* = 0.899; Figure 2). This is contrary to the findings of Gursoy et al. (2022), who conducted a retrospective review of medical records from 101 women spanning all rASRM stages and reported an inverse relationship between vitamin D and rASRM stage [45], and a meta-analysis by Qiu et al. (2020), which found that patients with endometriosis generally have lower vitamin D levels than controls, with some evidence linking deficiency to disease severity [46]. A systematic review by Kahlon et al. (2023) noted that this association varies across studies and often depends on the vitamin D status cut-off values used [23]. More recently, Farhangnia et al. (2024) concluded that, despite frequent vitamin D deficiency in severe cases, the overall relationship with disease severity remains inconclusive because of heterogeneous study populations and multiple confounders [4].

### 4.5. VDR Expression and Endometriosis Severity 

This is the first study to investigate the association between vitamin D receptor (VDR) expression and the severity of advanced stage III and IV endometriosis. The H-scores of stromal and epithelial VDR were comparable between stages III and IV (Table S5), and there was no association between the VDR H-score and rASRM score. Therefore, the endometriosis group was confined to stages III–IV, and both the rASRM stage III and vitamin D normal groups contained only four participants each, leading to a wide H-score dispersion and limited statistical power. However, Zhang et al. (2023) noted that tissue VDR measurements provide insights beyond those of serum vitamin D levels [47]. Previous studies have characterized VDR expression in endometriotic lesions (epithelium vs. stroma, lesion vs. normal endometrium); however, these findings have not been linked to serum vitamin D levels and clinical severity [11,19,41]. De Pascali et al. (2021) previously used semi-quantified VDR by IRS in both stromal and epithelial [19]. We applied qualitative and quantitative (H-score) methods because they are continuous and sensitive measures integrating staining intensity and positive cell percentage to improve the dynamic range and inter-observer concordance.

Moreover, the participants received heterogeneous treatment modalities, including various hormonal therapies that induce endometriotic cell atrophy and inhibit lesion proliferation, which can modulate VDR expression. Other factors that may modulate VDR expression beyond serum vitamin D and prior hormonal treatments [41] include VDR gene polymorphisms [20,21,22,23], and local immune responses [15]. Matasariu et al. (2023) reported that women pretreated with dienogest (2 mg daily for 3 months) showed higher mean serum 25(OH)D levels and concurrently lower epithelial VDR expression compared to the untreated group [41]. In contrast, we observed no significant differences in stromal or epithelial VDR H-scores among participants who received hormonal, nonhormonal, or no treatment. Nonetheless, variations in treatment duration or intervals between treatment and surgery could still have influenced VDR expression, emphasizing the need for further investigation (Table S3). 

### 4.6. Strengths and Limitations

This is the first study conducted in Thailand to investigate the relationship between vitamin D and endometriosis. Environmental exposure and dietary patterns have been identified as factors influencing the risk of developing endometriosis [34,35,48]. Country-specific data are essential, and differences in these factors across populations could partly explain the heterogeneous results reported globally. We adopted a matched case–control design, controlling for the two principal confounders of endometriosis—age and body mass index (BMI)—and subsequently performed multivariable-adjusted analyses for other variables that could influence disease occurrence. These methodological steps increased the internal validity and credibility of our findings. All participants with endometriosis underwent surgical evaluation with histopathological confirmation and were uniformly classified as rASRM stage III–IV, thereby creating an advanced-disease group without an insufficient number of less severe stages but enabling an accurate and precise assessment of serum vitamin D levels, tissue VDR expression, and clinical severity of the disease. Medical management of endometriosis may alter tissue architecture and staining patterns; to mitigate this, we performed comprehensive H&E staining and selected only those slides containing both stromal and epithelial components for VDR immunohistochemistry, ensuring consistent assessment of VDR expression to investigate how vitamin D functions through VDR, enabling active vitamin D to engage in cellular anti-inflammatory and immunomodulatory pathways. The H-score assessment was chosen because of its comprehensive continuous scale ranging from 0 to 300, which integrates quality (staining intensity) and quantity (percentage of positive cells) into a single value, offering a substantially wider dynamic range, and superior sensitivity to detect subtle gradations in staining intensity compared with the semi-quantitative immunoreactive score (IRS) used in previous endometriosis studies. This format is particularly suited for a detailed analysis to examine the relationship between serum 25(OH)D, VDR expression, and rASRM score. Evaluating VDR expression as a continuous variable rather than a dichotomous or four-level scale allowed us to detect a scored decrease in both stromal and epithelial VDR abundance in parallel with lower serum 25(OH)D, an effect that less detailed scoring systems may miss. 

Because this investigation was cross-sectional, vitamin D levels and disease status were captured simultaneously, and the temporal relationship between exposure and outcome remained indeterminate. Our study was adequately powered to detect differences in serum vitamin D levels as the primary outcome between the control and endometriosis groups; however, the overall sample size was insufficient to yield sufficient power to support reliable statistical analyses of the association between subgroup serum vitamin D status, VDR expression, and disease severity.

### 4.7. Clinical Implications and Future Directions

Notably, although our study found no significant difference in circulating vitamin D levels between the cases and controls, hypovitaminosis D is known to affect numerous health conditions; therefore, lifestyle optimization or vitamin D supplementation is likely to confer broader health benefits. This study highlights that many reproductive-aged Thai women exhibit hypovitaminosis D despite comparable occupational sunlight exposure (<50% outdoor work), implicating low vitamin D as a modifiable risk factor. The study’s findings may restrict the applicability of VDR as a biomarker for evaluating disease progression across various stages, as it exclusively included participants with advanced endometriosis. Future investigations should enroll both surgically and non-surgically managed patients to capture the complete rASRM spectrum, include women with serum total vitamin D ≥30 ng/mL, and carefully control for treatment modality and duration to boost statistical power and minimize confounders, thereby enabling in-depth analyses of serum 25(OH)D, VDR H-score, and disease severity. Longitudinal cohort studies should examine whether adjunctive vitamin D treatment initiated during pre-operative hormonal or non-hormonal therapy reduces symptom burden and slows stage progression of ovarian endometrioma, as well as the effects of vitamin D on different forms of endometriosis, including extragenital endometriosis. Such trials should also validate the VDR H-score as a prognostic biomarker and determine how supplementation modulates VDR expression in tissues. Clarifying these points will help determine whether restoring vitamin D sufficiency can modify the pathogenesis or progression of endometriosis and refine VDR-based risk stratification. 

## 5. Conclusions

In this study, no statistically significant associations were observed between serum total vitamin D levels, the presence of endometriosis, or disease severity. VDR expression was confined to the cytoplasm and was not associated with vitamin D levels or the severity of endometriosis. Similarly, no correlation was found between vitamin D levels and rASRM scores; however, it is important to note that the study included only participants with advanced-stage disease (stages III and IV). 

Given the limited sample size and the restriction to later stages of endometriosis, the possibility of a type II error cannot be excluded. Therefore, while our findings do not support a clear association, they do not preclude the potential role of vitamin D in modulating disease severity. This is particularly relevant in light of the tissue-level observations of VDR expression. Future research should investigate local vitamin D signaling pathways and VDR activity within endometriotic lesions, independent of systemic vitamin D status, to better understand their potential contributions to disease severity and progression.

## Figures and Tables

**Table 1:** Characteristics of endometriosis and the matching control group.

Characteristic	Endometriosis	Control	*p*-Value *
Age (years)	33.36 (5.41)	33.35 (5.63)	0.938
BMI (kg/m^2^)	21.76 [19.93, 23.19]	21.50 [19.24, 23.87]	0.927
BMI			0.841
○ Underweight	3 (8.33)	6 (8.33)	
○ Normal	23 (63.89)	46 (63.89)	
○ Overweight	3 (8.33)	6 (8.33)	
○ Obese I	7 (19.44)	14 (19.44)	
Living with a partner	25 (69.44)	47 (65.28)	0.668
○ Time with current partner (months)	48 [29, 108]	120 [68, 156]	0.024
Pregnancy history			
○ Pregnancy 1+	9 (25.00)	34 (47.22)	0.028
○ Abortion 1+	4 (11.11)	13 (18.06)	0.344
Contraception	3 (8.33)	29 (40.28)	0.002
Education			0.130
○ Undergraduate	5 (13.89)	19 (26.39)	
○ Graduate	31 (86.11)	53 (73.61)	
Occupation environment			
○ Outdoor working ≥50%	0	0	
○ Outdoor working <50%	36 (100.00)	72 (100.00)	
○ Total sun exposure (min/week)	115.00 [75.00, 150.00]	75.00 [47.50, 142.50]	0.304
○ Direct sun exposure (min/week)	74.30 [29.00, 109.00]	45 [00.00, 83.80]	0.133
○ Night shift working	3 (8.33)	10 (13.89)	0.469
Facial SPF			0.893
○ 0 (Non-user)	4 (11.11)	10 (13.89)	
○ 30	2 (5.56)	3 (4.17)	
○ 50	30 (83.33)	59 (81.94)	
Body SPF			0.310
○ 0 (Non-user)	8 (22.22)	29 (40.27)	
○ 15	8 (22.22)	8 (11.11)	
○ 30	3 (8.34)	4 (5.56)	
○ 50	17 (47.22)	31 (43.06)	
Outdoor exercise			0.334
○ None	32 (88.89)	62 (86.11)	
Consumption			
○ Meat product	34 (94.44)	72 (100.00)	>0.999
○ Dairy product	21 (58.33)	48 (66.67)	0.385
Residence			0.003
○ Urban	17 (47.22)	57 (79.17)	
○ Rural	19 (52.78)	15 (20.83)	

Data are expressed as mean (SD), median [interquartile range, IQR], or n (%). * Conditional logistic regression. Abbreviation: BMI, body mass index; SPF, sun protection factor.

**Table 2:** Comparison of vitamin D levels between endometriosis and a matching control group.

Vitamin D	Endometriosis	Control	Median Difference	(95% CI)
Unadjusted	20.45 [16.10, 25.10]	21.10 [18.20, 26.70]	–1.67	–4.16, 0.83
Adjusted model 1 *	20.11	23.01	−2.90	−5.26, −0.54
Adjusted model 2 ^†^	21.47	22.33	−0.86	−3.47, 1.75
Adjusted model 3 ^‡^	20.70	22.71	−2.01	−4.50, 0.48

Data are expressed as median [IQR]. * Model 1 was adjusted by residence and body sunscreen use—a generalized estimating equation. ^†^ Model 2 was adjusted by pregnancy and contraception. ^‡^ Model 3 was adjusted by residence, body sunscreen use, pregnancy, and contraception.

**Table 3:** Association between serum vitamin D status and endometriosis according to vitamin D deficiency in hypovitaminosis D subgroup.

Vitamin D	Endometriosis	Control	OR (95% CI)			
			Univariable	Multivariable		
			Crude	AOR Model 1 *	AOR Model 2 ^†^	AOR Model 3 ^‡^
≥20 ng/mL	19 (52.78)	40 (55.56)	1	1	1	1
<20 ng/mL	17 (47.22)	32 (44.44)	1.12 (0.45, 2.77)	1.99 (0.63, 6.35)	0.72 (0.24, 2.13)	1.28 (0.39, 4.16)

Data are expressed as n (%). * Model 1 was adjusted by residence and body sunscreen use—a conditional logistic regression. ^†^ Model 2 was adjusted by pregnancy and contraception. ^‡^ Model 3 was adjusted by residence, body sunscreen use, pregnancy, and contraception.

**Table 4:** Comparison of stromal and epithelial VDR expression (H-score) across vitamin D status categories in the endometriosis group.

Vitamin D	VDR H-Score Stroma	*p*-Value *	Difference (95% CI) ^†^	VDR H-Score Epithelial	*p*-Value *	Difference (95% CI) ^†^
Normal	81.25 (51.86)	0.876	Reference	190.63 (46.83)	0.908	Reference
Insufficient	77.13 (37.67)		−4.12 (−46.82, 38.59)	217.50 (69.45)		26.88 (−50.69, 104.44)
Deficient	68.57 (31.10)		−12.68 (−55.70, 30.35)	206.96 (67.83)		16.34 (−61.81, 94.49)
Severe deficiency	65.83 (44.18)		−15.42 (−73.38, 42.54)	206.67 (79.42)		16.04 (−89.24, 121.32)

Data are expressed as mean (SD). * One-way ANOVA. ^†^ Linear regression.

**Table 5:** Relationship between vitamin D status and severity in the endometriosis group.

Vitamin D	Endometriosis Severity		*p*-Value *	OR (95% CI) ^†^
	Stage 3	Stage 4		
Vitamin D status				
○ Normal	1 (2.78)	3 (8.33)	0.390	1
○ Hypovitaminosis D	3 (8.34)	29 (80.55)		3.22 (0.05, 57.99)
Hypovitaminosis D subgroup				
○ Insufficient	2 (5.56)	13 (36.11)		2.17 (0.14, 33.80)
○ Deficient	1 (2.78)	13 (36.11)		4.33 (0.24, 115.67)
○ Severe deficiency	0	3 (8.33)		-

Data are expressed as n (%) and mean (SD). * Fisher’s exact test. ^†^ Conditional logistic regression.

## Data Availability

The data supporting the findings of this study are available from the corresponding author upon reasonable requests.

## Supplementary Materials

The following supporting information can be downloaded at https://www.mdpi.com/article/10.3390/biomedicines13071605/s1, Table S1: Baseline characteristics based on vitamin D status; Table S2: Relationship between characteristic and risk of endometriosis; Table S3: Prior surgical management and treatment outcomes in the endometriosis group; Table S4: Association between serum vitamin D status and endometriosis according to subgroups of hypovitaminosis D; Table S5: Comparison of VDR expression (H-scores) in stromal and epithelial cells between stage 3 and 4 endometriosis; Table S6: Comparison of stromal and epithelial VDR expression (H-score) across prior treatments before surgery.

## Abbreviations

25(OH)D	25-Hydroxyvitamin D
AST	Aspartate aminotransferase
ALT	Alanine aminotransferase
BMI	Body mass index
COC	Combined oral contraception
Cu-IUD	Copper intrauterine device
C/S	Cesarean section
DMPA	Depot medroxyprogesterone acetate
GFR	Glomerular filtration rate
IHC	Immunohistochemical
TAH	Total abdominal hysterectomy
TR	Tubal resection
SPF	Sun protection factor
VAS	Visual analog scale
VDR	Vitamin D receptor
