# Supplementary material for: Analysis of Vitamin D and VDR Expression in Women with Advanced Endometriosis: A Case–Control Study in Thailand
Source: Biomedicines. 2025 Jun 30;13(7):1605. doi: 10.3390/biomedicines13071605 (PMC12292792; doi:10.3390/biomedicines13071605)
Supplement: Supplementary file 1 [file biomedicines-13-01605-s001.zip › biomedicines-3695063-Supplementary Materials.pdf]

Supplementary Materials

Table S1. Baseline characteristics based on vitamin D status.

Characteristics	Matched			Vitamin D status		
	Endometriosis n = 36	Control n = 72	p value*	Vitamin D ≥ 20 ng/mL n = 59	Vitamin D < 20 ng/mL n = 49	p value*
BMI			-			-
○ Underweight	3 (8.33)	6 (8.33)		4 (6.78)	5 (10.20)	
○ Normal	23 (63.89)	46 (63.89)		39 (66.10)	30 (61.23)	
○ Overweight	3 (8.33)	6 (8.33)		5 (8.47)	4 (8.16)	
○ Obese I	7 (19.44)	14 (19.44)		11 (18.64)	10 (20.41)	
Living with partner	25 (69.44)	47 (65.28)	0.668	43 (72.88)	29 (59.18)	0.111
Pregnancy history			0.028			0.125
○ Pregnancy = 0	27 (75.00)	38 (52.78)		31 (52.54)	34 (69.39)	
○ Pregnancy 1+	9 (25.00)	34 (47.22)		28 (47.46)	15 (30.61)	
Abortion history			0.344			0.264
○ Abortion = 0	32 (88.89)	59 (81.94)		50 (84.75)	41 (83.67)	
○ Abortion 1+	4 (11.11)	13 (18.06)		9 (15.25)	8 (16.33)	
Contraception			0.002			0.089
○ No	33 (91.67)	43 (59.72)		37 (62.71)	39 (79.59)	
○ Yes	3 (8.33)	29 (40.28)		22 (37.29)	10 (20.41)	
Contraceptive method						
○ COC	1 (2.78)	6 (8.33)				
○ DMPA	0	3 (4.17)				
○ Implantation	0	8 (11.11)				
○ Condom	0	4 (5.56)				
○ Cu-IUD	0	1 (1.39)				
○ TR	2 (5.55)	7 (9.72)				
Lower abdominal surgical history			0.055			0.016
○ No	25 (69.44)	61 (84.72)		44 (74.58)	42 (85.71)	
○ Yes	11 (30.56)	11 (15.28)		15 (25.42)	7 (14.29)	
Surgical procedure						
○ C/S	3 (8.33)	8 (11.11)				
○ Unilateral Ovarian Cystectomy	3 (8.33)	0				
○ Unilateral Salpingectomy	0	1 (1.39)				
○ Unilateral Salpingo-oophorectomy	2 (5.56)	0				
○ TAH	1 (2.78)	0				
○ Appendectomy	2 (5.56)	2 (2.78)				
Education			0.130			0.195
○ Undergraduate	5 (13.89)	19 (26.39)		16 (27.12)	8 (16.33)	
○ Graduate	31 (86.11)	53 (73.61)		43 (72.88)	41 (83.67)	
Occupation environment			-			
○ Outdoor working ≥ 50%	0	0				
○ Outdoor working < 50%	36 (100)	72 (100)		59 (100)	49 (100)	
Night shift working	3 (8.33)	10 (13.89)	0.469	7 (11.86)	6 (12.24)	1.000

 https://doi.org/10.3390/biomedicines13071605

Characteristics	Matched			Vitamin D status		
	Endometriosis<br>n = 36	Control<br>n = 72	p value*	Vitamin D<br>≥ 20 ng/mL<br>n = 59	Vitamin D<br>< 20 ng/mL<br>n = 49	p value*
Facial SPF			0.893			0.391
○ 0 (Non-user)	4 (11.11)	10 (13.89)		5 (8.47)	9 (18.37)	
○ 30	2 (5.56)	3 (4.17)		3 (5.08)	2 (4.08)	
○ 50	30 (83.33)	59 (81.94)		51 (86.44)	38 (77.55)	
Body SPF			0.310			<0.001
○ 0 (Non-user)	8 (22.22)	29 (40.27)		17 (28.81)	20 (40.82)	
○ 15	8 (22.22)	8 (11.11)		11 (18.64)	5 (10.20)	
○ 30	3 (8.34)	4 (5.56)		4 (6.78)	3 (6.12)	
○ 50	17 (47.22)	31 (43.06)		27 (45.76)	21 (42.86)	
Outdoor exercise			0.334			0.377
○ None	32 (88.89)	62 (86.11)		51 (86.44)	43 (87.76)	
○ < 120 min/week	4 (11.11)	4 (5.56)		4 (6.78)	4 (8.16)	
○ ≥ 120 min/week	0 (0.00)	6 (8.33)		4 (6.78)	2 (4.08)	
Outdoor exercise time						
○ Morning (06:00–10:00)	0	1 (1.39)				
○ Noon (10:00–14:00)	0	0				
○ Evening (14:00–19:00)	4 (11.11)	8 (11.11)				
○ Night (19:00–06:00)	0	1 (1.39)				
Consumption						
○ Meat products	34 (94.44)	72 (100.00)	-	58 (98.31)	48 (97.96)	-
○ Dairy products	21 (58.33)	48 (66.67)	0.385	36 (61.02)	33 (67.35)	1.000
Residence			0.003			0.234
○ Rural	19 (52.78)	15 (20.83)		23 (38.98)	11 (22.45)	
○ Urban	17 (47.22)	57 (79.17)		36 (61.02)	38 (77.55)	
Vitamin consumption			0.874			0.296
○ No	27 (75.00)	55 (76.39)		46 (77.97)	36 (73.47)	
○ Yes	9 (25.00)	17 (23.61)		13 (22.03)	13 (26.53)	
Vitamin product			0.874			
○ Vitamin B	3 (8.33)	1 (1.39)				
○ Vitamin C	5 (13.89)	14 (19.44)				
○ Ferrous	1 (1.39)	0				
○ Folic acid	1 (1.39)	1 (1.39)				
○ Collagen	2 (5.56)	1 (1.39)				
○ Fiber	0	1 (1.39)				
○ Protein	1 (1.39)	0				
○ CoQ10	1 (1.39)	0				
○ Glutathione	0	1 (1.39)				
○ Zinc	1 (1.39)	1 (1.39)				
Age (years)	33.36 (5.41)	33.35 (5.63)	0.938	33.07 (5.21)	33.69 (5.94)	0.879
BMI (kg/m^2)	21.76<br>[19.93, 23.19]	21.50<br>[19.24, 23.87]	0.927	21.81<br>[19.62, 23.79]	21.14<br>[19.23, 23.48]	0.644
Time with partner (month)	29.50 [0, 72]	64 [0, 126]	0.058	48 [0, 120]	30 [0, 120]	0.532
Occupation environment						

Characteristics	Matched			Vitamin D status		
	Endometriosis<br>n = 36	Control<br>n = 72	p value*	Vitamin D<br>≥ 20 ng/mL<br>n = 59	Vitamin D<br>< 20 ng/mL<br>n = 49	p value*
o Total sun exposure (min/week)	115.00 [75.00, 150.00]	75.00 [47.50, 142.50]	0.304	110.00 [60.00, 150.00]	75 [40.00, 125.00]	0.061
o Direct sun exposure (min/week)	74.25 [29.00, 109.00]	45 [00.00, 83.75]	0.133	50.00 [25.00,100.00]	31.50 [0.00, 90.00]	0.731
Laboratory results						
o Calcium (mg/dL)	9.42 (0.42)	9.41 (0.34)	0.897	9.45 (0.37)	9.36 (0.36)	0.332
o Magnesium (mg/dL)	2.15 (0.14)	2.16 (0.13)	0.601	2.17 (0.14)	2.14 (0.12)	0.637
o Phosphorus (mg/dL)	3.54 (0.49)	3.50 (0.44)	0.646	3.61 (0.47)	3.40 (0.43)	0.052
o Albumin (mg/dL)	4.40 [4.30, 4.60]	4.40 [4.30, 4.50]	0.822	4.4 [4.3, 4.6]	4.4 [4.3, 4.5]	0.961
o Creatinine (mg/dL)	0.76 (0.11)	0.73 (0.11)	0.152	0.74 (0.11)	0.74 (0.11)	0.783
o GFR (ml/min/1.73m^2)	104.59 (14.72)	107.17 (13.55)	0.348	105.50 (13.32)	107.28 (14.72)	0.362
o AST (IU/L)	17.50 [14.50, 20.50]	18.00 [15.00, 20.00]	0.830	18.00 [15.00,20.00]	17.00 [15.00, 20.00]	0.375
o ALT (IU/L)	14.00 [10.00, 16.50]	14.00 [12.00, 16.50]	0.619	15.00 [12.00, 17.00]	13.00 [10.00,16.00]	0.823

Data are expressed as mean (SD), median [interquartile range, IQR], and n (%). *Conditional logistic regression. Abbreviation: BMI, body mass index; COC, combined oral contraception; DMPA, depot medroxyprogesterone acetate; Cu-IUD; copper intrauterine device; TR, tubal resection; C/S, cesarean section; TAH, total pelvic hysterectomy; GFR, glomerular filtration rate; AST, aspartate aminotransferase; ALT, alanine aminotransferase; SPF, sun protection factor.

Table S2. Relationship between characteristic and risk of endometriosis.

Characteristics	OR	95% CI
Pregnancy history		
o Pregnancy 0	1	
o Pregnancy ≥1	0.41	0.19 – 0.91
Abortion history		
o Abortion 0	1	
o Abortion ≥1	0.54	0.15 – 1.93
Contraception		
o No	1	
o Yes	0.15	0.05 – 0.49
Lower abdominal surgical history		
o No	1	
o Yes	0.37	0.13 – 1.02
Vitamin consumption		
o No	1	
o Yes	1.09	0.39 – 3.02
Education		
o Undergraduate	1	
o Graduated	3.04	0.72 – 12.82
Night shift working		
o No	1	
o Yes	0.55	0.11 – 2.75

Characteristics	OR	95% CI
Facial SPF		
○ No	1	
○ Yes	1.27	0.34 – 4.69
Body SPF		
○ 0 (Non-user)	1	
○ Yes	2.32	0.91 – 5.94
Outdoor exercise		
○ No	1	
○ Yes	0.80	0.25 – 2.59
Consumption - Dairy products		
○ No	1	
○ Yes	0.72	0.34 – 1.51
Residence		
○ Rural	1	
○ Urban	0.20	0.07 – 0.57
Living with partner		
○ No	1	
○ Yes	1.19	0.54 – 2.65
Living with partner		
○ Time with partner (month)	0.99	0.99 – 1.00
Occupation environment		
○ Total sun exposure (min/week)	1.00	1.00 – 1.01
○ Direct sun exposure (min/week)	1.00	1.00 – 1.01
Laboratory result		
○ Calcium (mg/dL)	1.08	0.32 – 3.71
○ Magnesium (mg/dL)	0.38	0.01 – 14.74
○ Phosphorus (mg/dL)	1.19	0.56 – 2.55
○ Albumin (mg/dL)	0.78	0.09 – 6.70
○ Creatinine (mg/dL)	14.31	0.38 – 545.22
○ GFR (ml/min/1.73 m^2)	0.99	0.96 – 1.01

*Univariate conditional logistic regression. Abbreviation: SPF, sun protection factor.

Table S3. Prior surgical management and treatment outcomes in the endometriosis group.

Treantment history	Endometriosis
Type of treatment	
○ None	2 (5.56)
○ COC	1 (2.78)
○ DMPA	6 (16.67)
○ NSAIDs	13 (36.11)
○ GnRH agonist	7 (19.44)
○ Dienogest	7 (19.44)
Time of treatment (months)	9 [5, 12]
Pain (VAS, cm) *	5.40 [2.60, 8.60]
Satisfy (VAS, cm) †	6.92 (2.54)

Data are expressed as mean (SD), median [IQR], or n (%). *0: no pain, 5: moderate pain, 10: worst pain.; †0: very dissatisfied, 5: neutral, 10: very satisfied. Abbreviation: COC, combined oral contraception; DMPA, depot medroxyprogesterone acetate; NSAIDs, nonsteroidal anti-inflammatory drugs; GnRHa, gonadotropin-releasing hormone agonist; VAS, visual analogue scale.

Table S4. Association between serum vitamin D status and endometriosis according to subgroups of hypovitaminosis D.

Vitamin D	Endometriosis	Control	*p*-value	OR	95% CI
≥ 30 ng/mL	4 (11.11)	13 (18.06)	0.395	1	
< 30 ng/mL	32 (88.89)	59 (81.94)		1.84	(0.45, 7.57)
Subgroup					
20-29.99 ng/mL	15 (41.67)	27 (37.50)	0.842	1.54	(0.37, 6.49)
12-19.99 ng/mL	14 (38.89)	32 (44.44)		1.41	(0.32, 6.19)
< 12 ng/mL	3 (8.33)	0		-	-

Data are expressed as n (%). *Conditional logistic regression.

Table S5. Comparison of VDR expression (H-scores) in stromal and epithelial cells between stage 3 and 4 endometriosis.

	Endometriosis severity			
VDR expression	Stage 3	Stage 4	Difference (95% CI)*	*p*-value*
Stromal H-score	70.63 (22.95)	73.66 (37.57)	3.03 (-36.32, 42.39)	0.876
Epithelial H-score	198.75 [160, 223.75]	232.5 [165, 266.75]	29.25 (-55.00, 85.00)	0.443

Data are expressed as mean (SD), median [IQR] *Independent t-test.

Table S6. Comparison of stromal and epithelial VDR expression (H-score) across prior treatments before surgery

Treatment	n	VDR stromal H-score	*p*-value*	VDR epithelial H-score	*p*-value†
None	2	70 [57.5, 82.5]	0.778	246.25 (65.41)	0.694
COC	1	70		260	
DMPA	6	92.5 [77.5, 110]		184.17 (51.71)	
NSAIDs	13	60 [30, 87.5]		197.23 (79.88)	
GnRHa	7	95 [20, 95]		219.64 (55.70)	
Dienogest	7	70 [48.5, 105]		226.21 (61.20)	

Data are expressed as mean (SD), median [IQR]. *Kruskal-Wallis.; †One-way ANOVA. Abbreviation: COC, combined oral contraception; DMPA, depot medroxyprogesterone acetate; NSAIDs, nonsteroidal anti-inflammatory drugs; GnRHa, gonadotropin-releasing hormone agonist.
